# Enhanced immune response to hepatitis B vaccination through immunization with a Pre-S1/Pre-S2/S Vaccine

**DOI:** 10.1007/s00430-014-0374-x

**Published:** 2015-01-04

**Authors:** Daniel Shouval, Hedwig Roggendorf, Michael Roggendorf

**Affiliations:** 1 Liver Unit, Hadassah Hospital, Institute for Gastroenterology and Hepatology, Hadassah-Hebrew University, P.O. Box 12000, 91120 Jerusalem, Israel; 2 Institute for Molecular Immunology, Technical University of Munich, Munich, Germany; 3 Institute for Virology, Technical University of Munich, Munich, Germany

**Keywords:** Prevention of hepatitis B, Recombinant HBV vaccines, Immunogenicity, Sci-B-Vac™, Pre-S1/Pre-S2/S, Hepatitis B vaccine

## Abstract

Efficacy and safety of recombinant yeast-derived hepatitis B vaccines for prevention of hepatitis B have been demonstrated unequivocally worldwide as reflected in reduction in HBsAg carrier rates and hepatocellular carcinoma. A new generation of recombinant HBV vaccines expressed in mammalian cells containing Pre-S/S epitopes has been developed in several countries. Such vaccines are useful in special risk groups, i.e., in non-responders to conventional HBV vaccines including older adults, obese people, health care workers, patients with renal failure and on dialysis, transplant patients, patients with HIV as well as travelers on short notice to HBV endemic regions. The future of such vaccines depends on their enhanced immunogenicity and cost profile. Sci-B-Vac™ is a mammalian cell-derived recombinant Pre-S1/Pre-S2/S hepatitis B vaccine which has been shown to be highly immunogenic, inducing faster and higher seroprotection rates against HBV with higher anti-HBs levels at lower HBsAg doses as compared to conventional yeast-derived vaccines. Recently, it has been suggested that such Pre-S/S vaccines against HBV might be efficacious not only for prevention but also for intervention in persistent HBV infection. Data obtained in a recent clinical trial conducted in Vietnam in patients with chronic hepatitis B suggest that repeated monthly i.m. injections of the Sci-B-Vac™ co-administered with daily oral lamivudine treatment can suppress HBV replication and lead to anti-HBs seroconversion in ~50 % of treated patients. Optimization of protocols and efficacy of such an intervention, intended to bypass T cell exhaustion and immune tolerance to HBV remains to be explored.

## History of hepatitis B vaccines

First-generation, plasma-derived hepatitis B vaccines were developed in the US and France in the late 1970s. In the mid 1980s, second-generation recombinant DNA hepatitis B vaccines were constructed in yeasts transfected with HBV-DNA sequences coding for the small hepatitis B virus (HBV) surface protein (SHBs). These vaccines have gradually replaced the first-generation plasma-derived vaccines and are currently used for universal vaccination of newborns and adults in >170 countries worldwide [1–3]. Third-generation HBV vaccines containing one (Pre-S2) or two (Pre-S1 and Pre-S2) additional HBV envelope proteins have been developed in Germany, France, Korea and Israel in transfected mammalian cells [2, 4–6], Table 1 (see also article from W. Gerlich in this issue).

**Table 1:** Examples for three generations of hepatitis B vaccines

Vaccine type	Manufacturer	Envelope antigen	Remarks
Plasma derived	Hepatavax-B® (Merck & Co.)	SHBs	HBsAg, 5–40 μg/dose, licensed worldwide (not in use)
	Hevac B® (Pasteur M., France)	SHBsAg, (± MHBs)	HBsAg, 5–20 μg/dose, licensed in France (not in use)
	KGC® (Korea Green Cross)	SHBs^a^	HBsAg, licensed in East Asia
Recombinant, yeast derived	RECOMBIVAX® HB (Merck & Co., USA)	SHBs^a^	HBsAg, small S, 2.5–10 μg/dose, licensed worldwide
	Engerix-B® (GSK, Belgium)	SHBs^a^	HBsAg, small S, 10–20 μg/dose, licensed worldwide
	TGP 943™ (Takeda Chem, Japan)	SHBs^a^, MHBs	HBsAg (small S, Pre-S2) 10 μg/dose, licensed in Japan
Recombinant, mammalian cell derived	Gen Hevac B® (Pasteur M, France)^b^	SHBs, MHBs	HBsAg (S, Pre-S2), 20 μg/dose, licensed in France
	Sci-B-Vac/Bio-Hep-B™/Hepimmune™ (SciVac, Israel)^c^	SHBs, MHBs, LHBs	HBsAg, small S, Pre-S1, Pre-S2, 2.5–10 μg/dose, licensed in Israel and several countries in East Asia
	AG-3™ (Hepagene™) (Medeva, UK)^c^	SHBs, MHBs, LHBs	HBsAg, small S, Pre-S1, Pre-S2, 10–20 μg/dose, (not manufactured anymore)

^a^SHBs-p24

^b^Contain non-glycosylated p24 and glycosylated gp27, gp33, gp36

^c^Contain non-glycosylated p24 and p39, and glycosylated gp27, gp33, gp36, gp42

## Non-response to conventional hepatitis B vaccines and the rationale for development of a more immunogenic Pre-S/S hepatitis B vaccine

Successful seroprotection against HBV infection is defined by rate and by an anti-HBs titer of ≥10 mIU/ml following immunization or following resolution of HBV infection with the wild-type virus. Administration of three doses of licensed HBV vaccines to healthy subjects usually elicits an anti-HBs titer which is much higher than the defined seroprotection threshold of 10 mIU/ml and may reach several hundred to thousands of anti-HBs units. However, despite the extraordinary efficacy of second-generation HBV vaccines, immunization failure may occur and can sometimes be explained by variables such as improper storage, intra-gluteal injection, advanced age, obesity, renal failure, HIV infection, celiac disease, diabetes mellitus, chronic liver disease, inflammatory conditions such as Crohn’s disease, cancer and especially, immunosuppression [7–17]. Another important factor that may affect non-responsiveness to SHBs immunization seems to be genetically determined resistance [18, 19]. The ability to produce anti-HBs antibodies in response to immunization with the hepatitis B surface antigen (HBsAg) is controlled by autosomal dominantly expressed HLA Class II molecules. Milich et al. followed by Shouval et al. [6, 20] have shown in mice that non-responsiveness to immunization with small HBsAg (SHB) can be circumvented through immunization with Pre-S envelope proteins. Furthermore, immunization with synthetic middle Pre-S2 HBV peptide (MHBs) as well as with yeast and mammalian CHO cell-derived Pre-S2 vaccines may induce neutralizing antibodies and protect chimpanzees against HBV challenge [21]. Yet, initial experience in humans, using second-generation yeast-derived HBV vaccines containing mixtures of Pre-S and S antigens, did not lead to clinical application of such vaccines [22, 23]. Regardless of the initial fallback of Pre-S/S vaccines, there was and still is a justification for using more immunogenic vaccines that are efficacious in specific risk groups of non-responders or low responders to conventional HBV vaccination [24]. Such populations include genetic or advanced age-associated non-responders to yeast-derived vaccines (i.e., health care workers at risk, contacts of HBsAg carriers, newborns to HBsAg carrier mothers), patients with chronic liver disease or after liver transplantation, HIV patients, intravenous drug users, patients with chronic renal failure and on dialysis and other immunosuppressed patients. It was suggested that immunization with such Pre-S1/Pre-S2/S HBV vaccines may also reduce the risk of emerging of S gene mutants [25, 26].

## The development of a triple Pre-S1/Pre-S2/S hepatitis B vaccine

### Pre-clinical studies

Sci-B-Vac™ is an aluminum hydroxide adjuvanted recombinant hepatitis B vaccine, currently manufactured by SciVac Israel Ltd. The Pre-S1/Pre-S2/S vaccine was originally developed at the Weizmann Institute of Science, the Hadassah Medical Center and Biotechnology General in Israel. It was previously manufactured under the trade names Bio-Hep-B™ or Hepimmune™. It is produced in mammalian Chinese hamster ovary (CHO) cells, transfected with appropriate sequences that code for the HBV envelope proteins—the small S hepatitis B surface antigen (SHBs), the middle Pre-S2 (MHBs) and the large Pre-S1 envelope protein (LHBs) [27]. The gene coding for these antigens, including the native HBs promoter, enhancer and polyA signal, was cloned into a plasmid vector containing the mouse dihydrofolate reductase (DHFR) expression cassette. The plasmid was used to establish the producer CHO cell line. Transfected cells were selected for DHFR^+^ phenotype, and gene co-amplification was done with methotrexate. SDS-PAGE analysis of the purified HBs particles, which are of the *adw*_2_ subtype, secreted by the transfected CHO cells, revealed the presence of all three hepatitis surface antigens in non-glycosylated and glycosylated forms, mainly the SHBs (75–77 % p24, gp27), the MHBs (17–21 % gp33, gp36) and the LHBs (3–7 % p39, gp42) [6]. The *presence of* Pre-S2 and Pre-S1 antigens in the final vaccine preparation was confirmed in BALB/c mice and rabbits, which develop appropriate anti-Pre-S1 and anti-Pre-S2 antibodies following immunization with Sci-B-Vac™ [6]. The development of anti-Pre-S1 and anti-Pre-S2 antibodies and their relationship to anti-HBs levels were later also documented in immunized children [28–30]. The physical properties of the HBV envelope particles produced in CHO cells and the immune response to HBV Pre-S/S envelope proteins have been characterized in details [31, 32].

Comparative immunization studies in BALB/c mice revealed that the third-generation Pre-S1/Pre-S2/S vaccine has an extraordinary immunogenicity manifested by significantly higher seroconversion rates and higher anti-HBs titers in immune-competent animals immunized with Sci-B-Vac™, as compared to yeast-derived recombinant HBsAg vaccines such as H-B-Vax® and Engerix-B® [6]. Furthermore, the threshold immunogenic dose in 50 % of mice at day 14 after primary immunization was 0.13 μg for Sci-B-Vac™ and over sixfold higher for the other two yeast-derived control vaccines. Finally, and most importantly, mice of the strain B/10 M, which are genetically non-responsive to SHBs and MHBs antigens at the T cell level, were able to seroconvert following immunization with Sci-B-Vac™, with 100-fold higher anti-HBs titers as compared to conventional recombinant yeast-derived vaccines [6]. These results indicated that the CHO-derived vaccine elicits an augmented anti-HBs response in mice, as compared to yeast-derived vaccines, and is able to circumvent genetic resistance to small HBs.

### Clinical studies

To date, over 20 clinical studies have been completed in >3,000 patients immunized with Sci-B-Vac™ with an excellent safety record, including healthy adults, children and neonates [28–30, 33–39]. Additional ongoing studies are in progress in patients on hemodialysis and patients with HIV. In Israel, the Pre-S1/Pre-S2/S vaccine is in routine use for universal vaccination given on date of birth to ~50 % of all neonates (approximately 300,000 doses per year). The remaining 50 % of neonates currently receive Engerix-B®. Vaccine is usually administered at 0, 1 and 6 months, and formulations in alum hydroxide include 2.5 or 5 μg/injection of the envelope antigens for children up to 10 years old in non-endemic and endemic regions for HBV, respectively; 10 μg/injection for children >10 years of age and healthy adults and 20 μg/injection for immune-suppressed patients, patients on dialysis and for non-responders to conventional yeast-derived HBV vaccines.

**Fig. 1:**
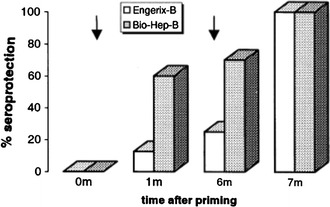
Rapid seroprotection against hepatitis B following the first dose of a Pre-S1/Pre-S2/S vaccine. Comparison of seroprotection (%), after one and two doses of 10 μg/1.0 ml of Sci-B-Vac (*n* = 10; *shaded bars*) and 20 μg/ml Engerix-B® (*n* = 8; *white bars*). *Arrows* reflect time of immunization. Reproduced by permission from Ref. [37]

In a pilot study conducted in medical students in Israel, the seroprotection rate defined as an anti-HBs titer of ≥10 mIU/ml following a single 10 μg dose of the Pre-S/S vaccine was already 70 % (mean anti-HBs titer of 81 mIU/ml) as measured at 6 months post-priming as compared to 25 % in the yeast-derived vaccinees immunized with 20 μg SHBs (mean anti-HBs titer of 12 mIU/ml), Fig. 1 [37]. After one booster injection at 6 months, 100 % seroprotection was achieved in all groups with anti-HBs levels rising 356-fold to 28,800 mIU/ml and 77-fold to 923 mIU/ml in recipients of Sci-B-Vac™ and Engerix-B®, respectively (*P* < 0.025). A second injection of Engerix-B® at 1 month in addition to the 6 month booster was required to reach similar anti-HBs levels for both groups of vaccinees at 7 months. From the early studies and onwards, it became evident that in comparison with yeast-derived HBV vaccines, >50 % of vaccinees (neonates, children and young adults) receiving Sci-B-Vac™ develop earlier seroprotection against HBV already within 4 weeks of a single (priming) dose.

A mathematical model was constructed to assess the pace and impact of anti-HBs production on immune memory following vaccination [40]. Wilson et al. collected data from 15 clinical trials, 14 of which monitored immune response to first-generation (plasma-derived) and second-generation (yeast-derived) HBV vaccines as well as trials conducted in recipients of third-generation vaccines, i.e., Sci-B-Vac™. The model predicts that third-generation Pre-S-containing vaccines induce immunity to HBV as early as 1–2 weeks from priming compared to the average 6–7 weeks in recipients of second-generation vaccines such as Engerix-B®, Recombivax® or Heberbiovac®.

The rapid immune response to Sci-B-Vac™ was corroborated by Schumann et al. [41] who recorded a fast and efficient cellular- and humoral-specific immune response against the envelope protein(s) of HBV using lymphocyte proliferation and Elispot assays which develops within a few weeks following the priming immunization.

**Fig. 2:**
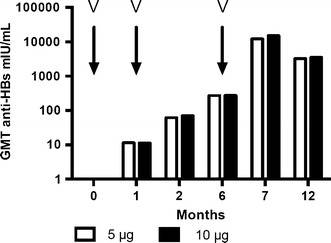
Anti-HBs response expressed as geometric mean titers (GMT), following immunization of vaccine and HBV naïve individuals with a Pre-S1/Pre-S2/S hepatitis B vaccine. Vaccinees (*n* = 105), 18–29 years old, M/F ratio 1:3, received three i.m. injections of Sci-B-Vac™ (V) at 5 or 10 μg/dose at 0,1 and 6 months. Seroprotection rates at 2, 6, 7 and 12 months post-priming were in the range of 79–83, 97–98, 100 and 100 %, respectively, for both groups. Quantitative anti-HBs response is expressed in mIU/ml. Modified, based on Ref. [35]

Several phase 2 and phase 3 studies were conducted in adults, children and neonates in Israel, Singapore, Vietnam, Philippines, Thailand and Poland [2, 28, 33–36, 38]. A few examples describing the main results of some of the studies are summarized as follows:

The enhanced immunogenicity induced through immunization with Sci-B-Vac™ was documented in a group of 105 young adults in Israel who reached 97 and 98 % seroprotection rates at month 6, following two injections of 5 or 10 μg/dose at 0 and 1 month, respectively. At month 7, 1 month after administration of a third injection, seroprotection rates were 100 % for both 5 and 10 μg doses, reaching an anti-HBs peak level of 12,177 and 14,998 mIU/ml, respectively. Thus, a mammalian cell-derived Pre-S/S vaccine dose of 5 μg was sufficient to induce an excellent humoral immune response, Fig. 2.

**Fig. 3:**
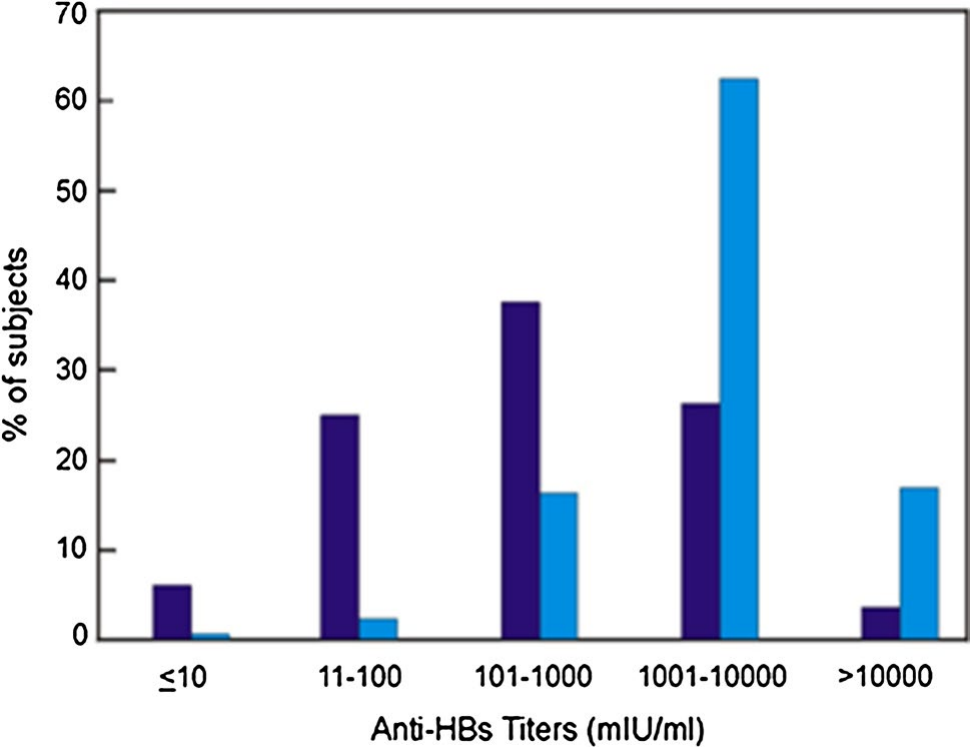
Distribution of anti-HBs titers in anti-HBc (-), HBsAg (-) neonates, immunized at birth and at 1 and 6 months, who received either 2.5 μg/0.5 ml, (*N* = 495; *dark blue bars*) or 5 μg/0.5 ml, (*N* = 200; *light blue bars*) Sci-B-Vac™/dose, data recorded at month 9 post-priming. (Data extracted from registration file, study HBN014-01)

In another study, conducted in neonates in Vietnam, a dose response relationship could be demonstrated using low doses of the Pre-S/S vaccine. More vaccinees who received the 5 µg dose/injection had anti-HBs titers ranging from 1,001 to 10,000 mIU/ml as compared to those who received the 2.5 µg dose/injection. More than 50 % of the vaccinees in the 5 µg dose group developed these high titers within 3 months after the third vaccination, Fig. 3. Furthermore, in study, HBN-014-01-2, 98.9 % had already protective anti-HBs titers at month 6 (following two vaccine doses) rising to 100 % at month 9, 3 months after injection of the third vaccine dose.

Cumulative data obtained during comparative immunogenicity studies were evaluated in larger cohorts containing populations from Israel as well as East Asia. On pooling data from two studies conducted in Singapore and Israel (protocols HBA 9006-S; 38-96-40), which compared the 10 μg Sci-B-Vac™ dose to the 20 μg Engerix-B® dose, the initial seroconversion rates at month 1 were significantly higher in the Sci-B-Vac™ group (67 %) than in the Engerix-B® group (19 %). GMT levels were significantly higher for Sci-B-Vac™ than Engerix-B® throughout the studies and peaked [5,999 mIU/ml for Sci-B-Vac™ and 2,056 mIU/ml for Engerix-B® (*P* = 0.001)] at 1 month following the third injection, respectively.

In comparative controlled studies conducted in Israel, more than 50 % of adult vaccinees who received the 10 µg/dose and neonates who received the 2.5 µg/dose had anti-HBs titers 100–10,000 mIU/ml after the third vaccination (Fig. 3). These rates were higher than those observed in vaccinees who received the yeast-derived control vaccines Engerix-B® and Hepavac II®. Furthermore, no non-responders were observed in immunized neonates who received 2.5 µg Sci-B-Vac™. In adults, the number of non-responders who received Sci-B-Vac™ was small (3.5 %, 18/513) as compared to vaccinees immunized with Engerix-B® (13.7 %, 43/415).

## The enhanced immunogenicity of the Pre-S1/Pre-S2/S vaccine in specific risk groups

### Patients with renal failure

Twenty-nine patients with end stage renal disease (ESRD) who did not respond in the past to repeated immunization with a double dose of a second-generation HBV vaccination protocol received 10 µg of Sci-B-Vac™ vaccine intramuscularly at 0, 1 and 6 months. Following immunization, 25/29 patients (86 %) developed seroprotective anti-HBs levels. In comparison, retrospective evaluation of the seroconversion rates in the same study center during the previous 3 years showed that only 19/36 (56.4 %) ESRD patients seroconverted using repeated immunization with a yeast-derived HBV vaccine [39]. This enhanced response was also expressed in a significantly higher titer of anti-HBs antibodies observed in the Sci-B-Vac™ cohort immunized with one-fourth of the HBsAg dose as compared to historical controls vaccinated with 40 µg/dose of yeast-derived HBsAg. The results of this pilot study require confirmation through a controlled comparative study which is due to start shortly.

### Non-responders to immunization with conventional yeast-derived HBV vaccines

A multicenter randomized comparative immunogenicity study of the Pre-S1/Pre-S2/S hepatitis B vaccine was conducted in eight European and Israeli centers [42]. The study population consisted of 716 individuals previously diagnosed as non-responders, mean age 50 years old, divided into two sub-cohorts of non-responders to 3 or 4 doses of a yeast-derived vaccine (anti-HBs titers <10 mIU/ml) and low responders to a conventional vaccination protocol (anti-HBs titer <100 mIU/ml). Study participants classified as non-responders or low responders received a first dose of Sci-B-Vac™ at 20 μg on day 0. If the elicited anti-HBs titer at day 36 ± 7 was <100 mIU/ml, participants received a second booster on day 85 ± 7. Engerix-B®, at a dose of 20 μg/injection, was used as a control vaccine. In the primary study population (non-responders after ≥4 previous injections, ITT analysis) at 1 month after the first or second injection, the proportion of vaccinees with anti-HBs titers of ≥100 IU/l was higher for those injected with Sci-B-Vac™ (35.7 %) as compared to the conventional control vaccine (20.8 %; *P* = 0.006). Using the threshold for seroprotection of ≥10 mIU/ml anti-HBs, the results showed an even higher response rate for the Pre-S1/Pre-S2/S vaccine as compared with the S vaccine (81.7 and 49.1 %, respectively; *P* < 0.001). These data which included HLA typing confirmed the initial observation in non-responder mice that genetically determined non-response to conventional yeast-derived HBV vaccines can be partially overcome through immunization with a mammalian cell-derived triple antigen Pre-S1/Pre-S2/S vaccine. Thus, the study by Rendi-Wagner et al. [42] established that two injections of this third-generation vaccine to be superior to conventional vaccines in inducing relatively high (≥100 IU/l) anti-HBs antibody titers in both non-responders and low responders to previous conventional vaccination. Furthermore, it also confirmed the preliminary observation from Singapore regarding effectiveness of this vaccine in non-responders to first-generation, plasma-derived HBV vaccines [43].

### Patients with overweight

**Fig. 4:**
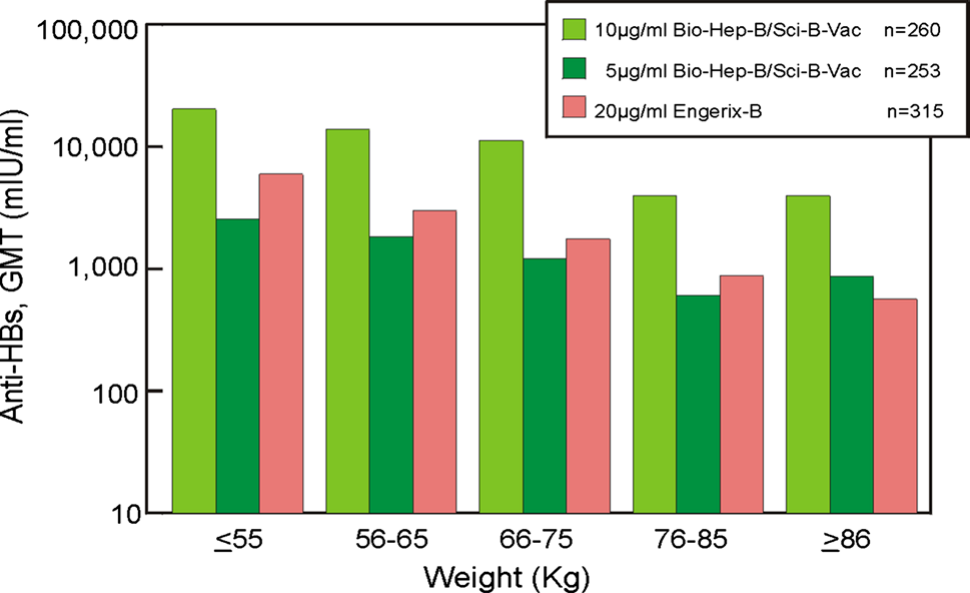
Effect of body weight on immunogenicity. Comparison of GMT (anti-HBs titers) in adults who received three doses of either Sci-B-Vac™ (Bio-Hep-B™) or Engerix-B® at 0, 1 and 6 months (studies 38-92-001 and 38-96-040), measured at month 7). Reproduced from the registration file of Sci-B-Vac™ in Israel

Increased body weight is one of a number of factors associated with reduced immunogenicity of HBV vaccines. Figure 4 demonstrates the effect of weight on quantitative anti-HBs levels in adult vaccinees in studies 38-92-001 and 38-96-040. In these comparative controlled trials, increased body weight was associated with a decrease in seroprotection rates in recipients of yeast-derived vaccines. In contrast, the impact of overweight on anti-HBs levels was practically neutralized in adults immunized with three doses of Sci-B-Vac™ at 10 µg/dose as compared to recipients of a yeast-derived vaccine Engerix-B® who received a 20 µg/injection, twice the dose of Sci-B-Vac™ (*P* < 0.012), Fig. 4. These data confirm a similar observation from Japan where high weight Sumo wrestlers responded more favorably to a Pre-S2/S HBV vaccine as compared to a conventional yeast-derived S-containing vaccine (Yamada K et al. Hepatology Research 1998; 12:3, abs).

### Immune-suppressed patients

The enhanced immunogenicity of the Pre-S1/Pre-S2/S HBV vaccine under conditions of immune suppression was demonstrated in a number of settings. In a study by Lo and co-workers from Hong-Kong, 20 patients who underwent liver transplantation for chronic HBV infection treated with lamivudine, but not with hepatitis B immune globulin (HBIG), received two courses each of three double doses (20 µg) of Sci-B-Vac™ at a median of 637 days after transplantation [44]. Five patients (25 %) responded to the first vaccination course and five additional patients responded after the second course with an overall response rate of 50 %. The response rate was 88 % in patients <50 years old and 25 % in older patients (*P* = 0.02). The median peak anti-HBs titer was 153 mIU/ml with six responders having a titer >100 mIU/ml and seven had a sustained response >6 months. Among seven previous non-responders to second-generation recombinant vaccine, 3 (44 %) responded. In conclusion, immunization with Sci-B-Vac™ was effective in about 50 % of immunosuppressed, HBV-infected liver transplant patients receiving lamivudine prophylaxis without HBIG.

A similar experience was recently reported from Germany where an obese 40-year-old pregnant woman developed fulminant hepatitis and underwent liver transplantation [45]. The patient was treated with lamivudine and HBIG and 3 years after transplantation was immunized with five doses of Sci-B-Vac™ at an 1–2 week interval without an adequate anti-HBs response. Three months later, she was re-vaccinated with three additional doses of the Pre-S1/Pre-S2/S vaccine which led to anti-HBs seroconversion. Once HBV-specific lymphocyte proliferation reached a stimulation index of 8.7, with a corresponding HBV-specific enzyme-linked immunosorbent spot assay response, HBIG treatment was withdrawn, 3 months after initiation of vaccination. Anti-HBs titer remained >700 mIU/ml for at least 6 months. These results provide further support to the conclusion that the Pre-S1/Pre-S2/S HBV vaccine can break tolerance to HBV envelope proteins even under conditions of heavy immune suppression induced by combined treatment of tacrolimus, mycophenolate mofetil and prednisone.

**Fig. 5:**
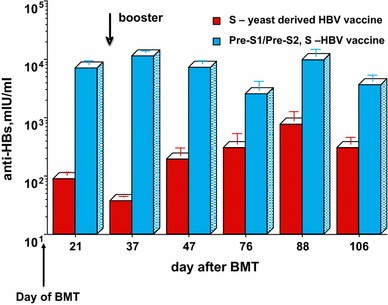
Adoptive transfer of immunity to HBV in mice: anti-HBs response in immune-suppressed BALB/c mice (*N* = 10), treated by whole body irradiation and bone marrow transplantation from immune-competent donor mice immunized i.p. with 1 μg of HBsAg (D. Shouval, unpublished data)

Further support for the enhanced immunogenicity of the Pre-S/S HBV vaccine was demonstrated by our group in irradiated, immune-suppressed BALB/c mice transplanted with bone marrow (BMT) from matched donor mice, immunized with 1 μg of either Sci-B-Vac™ or a yeast-derived HBV vaccine i.p. [46]. BMT recipient mice developed protective anti-HBs titers within days of BMT from immunized donors [46]. Moreover, BMT recipient mice from Pre-S1/Pre-S2/S-immunized donors developed significantly higher anti-HBs titers as compared to mice transplanted with bone marrow obtained from mice immunized with a yeast-derived control HBV vaccine (D. Shouval, unpublished results), Fig. 5.

The ability to induce protective anti-HBs antibody levels in immune-suppressed organ transplant recipients through adoptive transfer of immunity was further confirmed in other animal models such as rats and woodchucks [47, 48].

Indeed, active transfer of humoral and cellular immunity through adoptive transfer of immunity to HBV was also repeatedly demonstrated in HBV naïve human BMT recipients transplanted with immune cells from a vaccinated donor as well as in BMT recipients with persistent HBV infection transplanted with an HBV immune bone marrow [46, 49–52]. In a pioneering study, Schumann et al. [53] immunized 14 potential live liver donors with repeated double doses of Sci-B-Vac™ and followed the development of cellular and humoral immunity in their liver transplant recipient. Adoptive transfer of cellular and/or humoral immunity to HBsAg was demonstrated in three liver transplant recipients by lymphocyte proliferation and Elispot assays as well as by conventional anti-HBs immunoassay. The intensity of the humoral immune response in liver transplant recipients was proportional to the anti-HBs levels generated in donors through immunization with Sci-B-Vac™. These investigators have also shown a similar phenomenon in liver transplant patients with persistent HBV infection who received a liver lobe from an immunized donor with temporal clearance of circulating, peripheral HBV-DNA.

## Prospects of Sci-B-Vac™ as a therapeutic vaccine

Currently available anti-viral agents against persistent HBV infection include interferon alpha as well as nucleotide and nucleoside analogues (NUCS). Only a minority of preselected patients reach a sustained response to interferon. NUCs are quite effective in suppression of viraemia and intra-hepatic viral replication but have no direct impact on intra-nuclear, covalently closed circular cccHBV-DNA which can persist for years and serves as template for the transcription of HBV pregenomic RNA [54]. It remains to be seen if the recent detection of sodium taurocholate co-transporting polypeptide identified as the HBV receptor [55] will lead to development of more efficient anti-viral agents which support elimination of cccHBV-DNA by preventing infection of new hepatocytes [56].

Most patients persistently infected with HBV are either immunotolerant or immunologically hyporesponsive, displaying a weak cellular and altered cytokine immune responses to viral nucleocapsid and envelope antigens [57]. During the past 15 years, repeated attempts have been made to develop immunologic and pharmacologic means to resolve the well described immunologic hyporesponsiveness and T cell exhaustion to HBV proteins in such patients [57]. These include among others repeated immunization with conventional S vaccines and development of more immunogenic vaccines such as Pre-S2/S [5, 58, 59], Pre-S1/Pre-S2/S vaccines [3, 6], vaccines containing HBsAg-anti-HBs immune complexes [60, 61], lipopeptide-based vaccines [62] and DNA vaccines [63]. Furthermore, other means for stimulating an enhanced immune response against HBV include development of new adjuvants with improved immune stimulation as compared to the conventional aluminum hydroxide used in most vaccines or using an intradermal route for vaccine administration (reviewed in [24, 26, 64–70]). The use of conventional or modified HBV vaccines for intervention in persistent infection (in contrast to prevention) has been termed “therapeutic vaccination” intended to restore dysfunctional immune and in particular T cell responses. Cumulative data collected during the past decade suggest that the utility of such immunologic maneuvers has met with limited success [65]. For example, already in 1994, Pol et al. [71] have conducted a pilot study immunizing HBsAg carriers with the mammalian cell-derived Pre-S2/S HBV vaccine GenHevac B® and observed a ~50 % reduction in viral replication. However, a randomized controlled trial in recipients of 20 μg/dose GenHevac B®, and two control groups receiving either yeast-derived Recombivax® or no treatment did not confirm the initial observations [5]. Yet, HBV-DNA “negativization” rates were somewhat higher in the Pre-S2/S vaccine recipient (15 %) as compared to the control group (2.7 %), but this effect was not sustained following cessation of vaccination. Regardless of several failures in using GenHevac B® as a therapeutic vaccine [5, 72], these investigators could demonstrate a specific CD4 T cell response against viral envelope epitopes in some of the vaccinees [58]. A similar observation was reported from Japan where Ren et al. [73] evaluated a transfected hepatoma cell line-derived product containing glycosylated and non-glycosylated SHBs with a small amount of MHBs. Combining SHBs with more potent adjuvants than alum hydroxide such as AS02 [74] or immunization with HBsAg-anti-HBs immune complexes [75] did not prove so far to be significantly effective in clearing persistent HBV infection. Results obtained in some of these studies suggest that success of immunotherapy through vaccination may be inversely proportional to the degree of viral load. Consequently, effective suppression of viral load before or at least at initiation of vaccinotherapy seems logical. Boni et al. [76] have shown that high concentration of HBV antigens can disrupt T cell functions which can be restored in vitro through suppression of viral load by NUCs such as lamivudine or entecavir. Indeed, lamivudine treatment can restore immunologic hyporesponsiveness in patients with chronic hepatitis B (CHB) through enhancement of a CD4-mediated response to nucleocapsid antigens within 4–7 days of treatment initiation [77]. However, such stimulation and restoration of T cell reactivity seem to be transient. Therefore, immune stimulation of the hyporesponsive HBV-specific T cells through therapeutic vaccination should be initiated early after initiation of anti-viral treatment with lamivudine [78].

A few years ago, an attempt was made to assess the immunomodulatory impact of oral administration of HBsAg on the cellular and humoral immune response against HBV surface proteins [79]. Feeding of mice with HBV surface antigens followed by immunization with the Pre-S/S HBV vaccine BioHep B lead to significant suppression of anti-HBs antibody levels. The investigators concluded that oral tolerance induction effectively down-regulated pre-existing immunity and reduced the anti-HBs titers in previously immunized mice to 112 versus 223 mIU/ml, in tolerized compared with non-tolerized controls (*P* < 0.01). These results obtained in mice are difficult to reconcile with data obtained in a follow-up non-controlled study in humans who received oral treatment with Sci-B-Vac™ (without aluminum hydroxide). Feeding 42 patients with persistent HBV infection was associated with a 26 % HBeAg seroconversion rate during a 20-week follow-up period with a decrease in viral load in 36 % of recipients. This effect was also reported to be associated with augmentation of HBsAg-specific T cell proliferation, interferon gamma positive T cell clones and natural killer T cell activity in >50 % of patients [80]. These results contradict the current understanding of the immunopathology of persistent HBV infection and require confirmation using adequate controls including groups to be fed with placebo as well as with HBcAg. Until then, the implications of these results as such are open to different interpretations.

Previous observations suggest that conventional and Pre-S2/S HBV recombinant HBV vaccines may induce some suppression of viral load in patients with persistent HBV infection which seems more effective in the presence of low-level viraemia. Based on the cumulative experience, Hoa et al. evaluated the potential of using Sci-B-Vac™ as a therapeutic vaccine in patients with CHB. In this controlled clinical trial conducted in Vietnam between 2003 and 2006, 180 HBsAg+/HBeAg+ CHB patients, 16–70 years old, were randomized to receive 8 monthly i.m. doses of Sci-B-Vac™ 20 μg/dose alone (VAC), 8 monthly i.m. injections of Sci-B-Vac™ 20 μg/dose in combination with 100 mg lamivudine daily (VAC + LAM) or monotherapy with lamivudine 100 mg/day (LAM). Fourteen patients discontinued treatment prematurely. The main results of this study include:

1. Significant suppression of median viral load from an overall base line of ~10^6^ log copies/ml by at least 1 log copy/ml was observed at 3 months for the VAC + LAM and the LAM treatment groups being maximal for the combination group at a 65 % response rate (median reduction of viral load 2.6 log copies/ml) as compared to 55 % in the LAM group (1.9 log copies/ml) and 18.3 % (1 log copies/ml) in the VAC group, Fig. 6. Furthermore, an overall rate of HBV-DNA suppression <4 log copies/ml was observed in 55 % of the VAC + LAM group as compared to 28.3 % in the monotherapy with LAM. This effect was sustained and still present at month 12, 4 months after administration of the last vaccine dose. These results suggest that co-administration of Sci-B-Vac™ and lamivudine have a synergistic effect on suppression of viral load as compared to vaccination alone or monotherapy with lamivudine. At month 18 post-initiation of treatment, viral load suppression was still documented in 51.7 and 43.4 % of VAC + LAM and LAM recipients, respectively. Yet, from the data presented, it seems that the enhanced viral suppression afforded by administration of Sci-B-Vac™ in the combination group was waning at month 18, when patients were off treatment for 10 months.

**Fig. 6:**
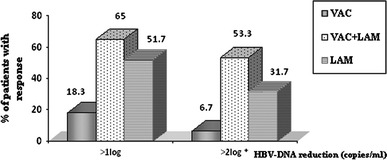
HBV-DNA reductions at 3 months post-treatment. The percentage of patients with early HBV-DNA reduction (after 3 months) was expressed using two cutoff levels (reductions of >1 log copy/ml and >2 log copies/ml). The *asterisk* indicates a significant difference between the VAC + LAM and LAM groups (*P* < 0.05). VAC-recipients of Sci-B-Vac™, V + L-recipients of Sci-B-Vac™ and Lamivudine, LAM recipients of lamivudine. Reproduced by permission Ref. [81]
2. At month 18, 10 month after cessation of treatment, seroconversion rates to anti-HBs ≥10 mIU/ml were observed in 51.7 % of the combination group, in 31.7 % of recipients of vaccine alone and in none of the lamivudine recipients. Vaccine-induced anti-HBs responses ≥10 mIU/ml were already documented at month 9, 1 month after the last vaccine dose in both the VAC and VAC + LAM recipients at 21.7 and 35 %, respectively, rising to 28.3 and 45 % at month 12, respectively. Furthermore, at month 12, 4 months after the last vaccine injection, anti-HBs levels between 100 and 1,000 mIU/ml were documented in 49 % of all vaccine recipients regardless of study group, Table 2.
3. HBsAg loss was documented in only one patient in the VAC group, in two patients in the VAC + LAM recipients and in one LAM treated patient. HBeAg loss was similar in all three treatment arms ranging between 21 and 33 % at month 18. Sero-reversion to HBeAg positivity was documented at the end of the observation period in 6.7 and 5.0 % of VAC + LAM and LAM recipients, respectively. Biochemical response defined as alanine amino transferase (ALT) reduction to <1.5 upper limit of normal levels was reported for 63, 67 and 78 % of VAC, VAC + LAM and LAM recipients, respectively, *P* non-significant.
4. Although a more detailed description of the safety follow-up is not presented in the report of Hoa et al. [81], the authors state that they did not observe any serious adverse events or hepatitis flares during the study period.

**Table 2:** Rates of vaccine-induced anti-HBs seropositivity for the three groups after eight i.m. injections of 20 μg/dose Sci-B-Vac™ as tested at month 18, 10, months after administration of the last treatment. VAC-recipients of Sci-B-Vac™, V + L-recipients of Sci-B-Vac™ and Lamivudine, LAM recipients of lamivudine. Reproduced by permission from Ref. [81]

Mo	No. (%) of anti-HBs responders in the following treatment group			*P* ^*a*^
	VAC	V + L	LAM	
3	2 (3.3)	3 (5.0)	0	NS
9	13 (21.7)	21 (35.0)	0	<0.001
12	17 (28.3)^b^	27 (45.0)^b^	0	<0.001
15	17 (28.3)^b^	28 (46.7)^b^	0	<0.001
18	19 (31.7)^b^	31 (51.7)^b^	0	<0.001

^a^For the overall treatment effect

^b^The difference between the VAC and V + L groups was significant (*P* < 0.05)

Analysis of the data of the clinical trial conducted by Hoa et al. [71] suggest that, as previously observed with another PreS-2/S vaccine, such vaccines are able to induce some suppression of viral load through a yet unknown mechanism, an effect which as shown in the present study, can be augmented by NUCS, i.e., lamivudine. Furthermore, co-administration of Sci-B-Vac™ with lamivudine lead to an unprecedented anti-HBs seroconversion rate of about 50 % which seem to contribute to the control of persistent infection as also reflected in improvement of markers for hepatocellular injury. These data therefore suggest that despite previous doubts regarding the ability of aluminum adjuvanted vaccines to restore the immunologic T cell-associated hyporesponsiveness, repeated immunization with a highly immunogenic Pre-S1/Pre-S2/S vaccine can bypass immune tolerance at least in some patients. However, the study protocol and results had a number of limitations and several issues regarding the utilization of Sci-B-Vac™ and co-administration with a nucleos(t)ide analogue for vaccinotherapy require further study. Further information is required regarding the durability of anti-HBs levels with precise quantitative anti-HBs and HBV-DNA monitoring under treatment. Use of more potent NUCs than lamivudine as well as assessment of intra-hepatic cccDNA and the requirement for further vaccine booster injections should also be assessed. Further studies are also required regarding the monitoring of the adoptive and innate immune response in responders and non-responders to vaccinotherapy.

Since the discovery of HBsAg as a hepatitis-associated antigen in 1968 [82], eradication of persistent HBV infection has been an elusive goal. The definition of cure has changed over the past decades as it became clear that suppression of viral replication, HBeAg seroconversion and even HBsAg seroconversion to anti-HBs are not sufficient to clear the intra-hepatic cccHBV-DNA and prevent dividing hepatocytes from new HBV infection [54]. Consequently, the goal of clearing persistent HBV infection was put at least temporarily on “hold” while immunologic control of HBV appeared a more achievable target. Indeed, repeated injections with a highly immunogenic Pre-S1/Pre-S2/S vaccine seem to induce an 18 months sustainable anti-HBs response with corresponding partial control of circulating HBV-DNA.

## Abbreviations

Anti-HBs: Antibodies to HBsAg
ALT: Alanine amino transferase
BMT: Bone marrow transplantation
cccHBV-DNA: Covalently closed circular HBV-DNA
CHB: Chronic hepatitis B
CHO: Chinese hamster ovary cells
DHFR: Dihydrofolate reductase
ESRD: End stage renal disease
HBIG: Hepatitis B immune globulin
HBV: Hepatitis B virus
HBcAg: Hepatitis B core antigen
HBeAg: Hepatitis B e antigen
HBsAg: Hepatitis B surface antigen
ITT: Intention to treat analysis
LAM: Lamivudine
LHBs: Large hepatitis B surface antigen (Pre-S1)
NUCS: Nucleos(t)ide analogues
mIU: Milli International Units
MHBs: Middle hepatitis B surface antigen (Pre-S2)
SHBs: Small hepatitis B surface antigen (S)
VAC: Vaccine
